# Rapidly progressive necrotizing pneumonia: remember the *Streptococcus anginosus* group!

**DOI:** 10.11604/pamj.2020.36.116.22218

**Published:** 2020-06-23

**Authors:** Biplab Kumar Saha

**Affiliations:** 1 Ozarks Medical Center, West Plains, Missouri, United States of America

**Keywords:** Necrotizing pneumonia, lung abscess, pyopneumothorax, *Streptococcus anginosus* group

## Abstract

Acute necrotizing pneumonia in an immunocompetent host is uncommon and usually caused by *Staphylococcus aureus* infection. *Streptococcus anginosus* group (SAG) is a less recognized cause of rapidly destructive lung infection resulting in significant patient morbidity and mortality. Unlike many other bacterial infections, SAG can cross fascial planes and cause fulminant infections. Necrotizing pneumonia and lung abscesses due to SAG often fails conservative therapy with antimicrobials and requires definitive surgical intervention. Consideration of SAG as a potential etiology might help to institute definitive therapy earlier and prevent complications.

## Introduction

Acute necrotizing pneumonia in an immunocompetent host is an uncommon complication of bacterial pneumonia. Most cases of community-acquired necrotizing pneumonia are secondary to *Staphylococcus* infection, especially in patients who have suffered or are suffering from influenza or parainfluenza infection. Although reported in the literature, the *streptococcus anginosus* group (SAG) is not often considered in the differential diagnosis of necrotizing parenchymal lung infection. SAG is a normal flora of the oral cavity and is comprised of 3 distinct species: *S. anginosus, S. constellatus* and *S. imdermedius*. Infection with these bacteria could be extremely aggressive with a rapid formation of lung abscess and or empyema. Due to their ability to cross the fascial plane, the infection could erode through the chest wall or spill into the mediastinum resulting in fulminant mediastinitis, sepsis, and shock state. In contrast to an abscess in other bodily organs, lung abscess, historically is not drained by surgical or percutaneous interventions. As the communication of the abscess cavity with the airway results in ‘auto drainage’ of the abscess, a drainage procedure is considered unnecessary or potentially dangerous. Long-term antibiotic therapy with anaerobic coverage is the standard of care. However, in patients who develop lung abscess or empyema due to SAG infection, drainage and possibly surgical resection is essential in addition to the antimicrobial therapy for resolution of the infection. Delay in timely care could be life-threatening and causes significant morbidity.

## Patient and observation

A 45-year old male presented to the ED with mild fever, sneezing, rhinorrhea, cough, right-sided dull chest pain and shortness of breath. He was recently exposed to a co-worker suffering from ‘flu’ and started experiencing upper respiratory tract infection symptoms two days ago. He was nauseous, vomited several times and suffered from diarrhea. The persistent dull chest pain and worsening exertional shortness of breath for a day prompted the ED visit. His past medical history was significant for Crohn´s disease for which he was not on any medication. The patient was a smoker of about half a pack per day and denied any history of alcohol or recreational drug abuse. He traveled to South Africa twenty-three years ago and didn´t have any pet at home. No personal history of tuberculosis (TB) or known exposure to anyone with TB. He worked at a fast-food restaurant and did not have any family history of vasculitis or lung cancer. Vital signs on admission were, a blood pressure of 135/89 mmHg, a pulse of 124 beats per minutes, a temperature of 37.8°C, a respiratory rate of 23 breaths per minute and oxygen saturation of 94% on 5L oxygen via nasal cannula. Physical examination showed a young man in mild distress from tachypnea.

**Figure 1:**
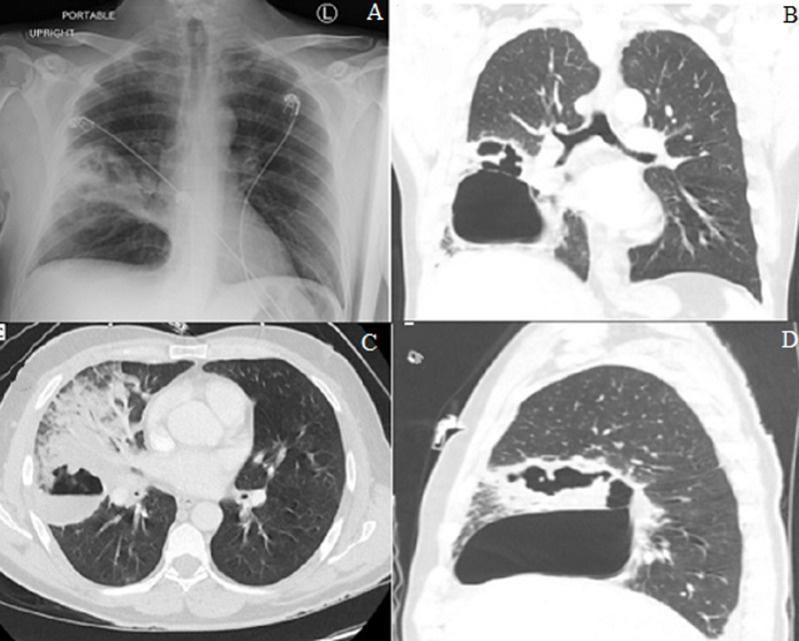
A) chest X-ray on admission showing focal infiltrate in the right midlung zone involving the minor fissure with multiple areas of lucencies suggestive of abscess formation; B) computed tomography (CT) axial view demonstrating dense consolidation of the right middle lobe (RML) and abscess with air-fluid level; C) coronal CT with revealing RML necrotizing pneumonia as well as thick wall cavity in the right lower lobe (RLL); D) C) sagittal view with RML and RLL necrotizing pneumonia and abscess formation

He appeared dehydrated. Chest auscultation revealed bronchial breath sound and coarse crackles in the right mid and lower lung zone, both anteriorly and posteriorly. The rest of the examination was normal. Blood work was significant for leukocytosis, 27.1 x10^3^/micro L with neutrophilia of 88% and bandemia of 3%. The rest of the complete blood count and comprehensive metabolic panel were normal. His lactic acid was 1.5mmol/L, and procalcitonin level was elevated at 0.69ng/ml. The chest X-ray is shown in Figure 1. A computed tomography (CT) of the chest was performed and representative slices are shown in Figure 1. The respiratory viral panel was positive for coronavirus. Sputum gram stain and culture showed normal respiratory flora. Urinary antigen testing for legionella and pneumococcus were negative. A workup for vasculitic disorders was negative. HIV antibody test was also negative. A diagnosis of necrotizing pneumonia due to secondary bacterial infection in the setting of coronavirus infection was made. *Staphylococcus aureus* (SA) was strongly suspected to be the etiologic agent. However, the patient was less toxic appearing than what would be expected with SA necrotizing pneumonia and a subacute vasculitic disorder, like granulomatosis with polyangiitis, was also considered.

**Figure 2:**
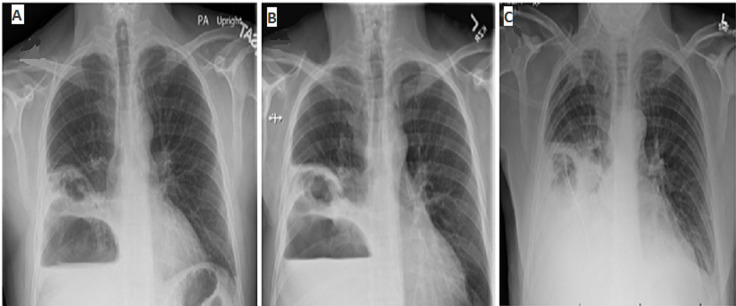
A) day 1 post-admission chest X-rays showing the development of large fluid-filled cavities; B) day 2 post-admission chest X-rays showing the development of large fluid-filled cavities; C) reduction in the airspace in the right hemithorax (day 3)

**Figure 3:**
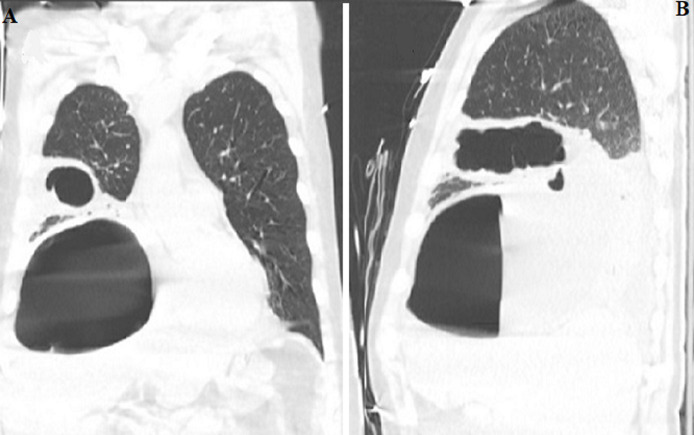
A) coronal chest CT on day 3 showing a significant increase in the size of the abscess cavity and dense consolidation in RLL; B) sagittal chest CT on day 3 showing a significant increase in the size of the abscess cavity and dense consolidation in RLL

**Figure 4:**
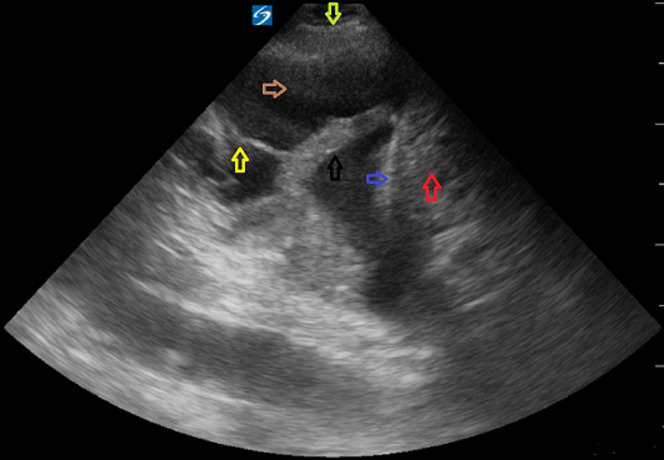
bedside ultrasound showing complex pleural effusion with cellularity (brown arrow), septations (yellow arrow) and atelectatic lung (black arrow). The diaphragm (indigo arrow), liver (red arrow), chest wall (lime arrow) are also visible

**Figure 5:**
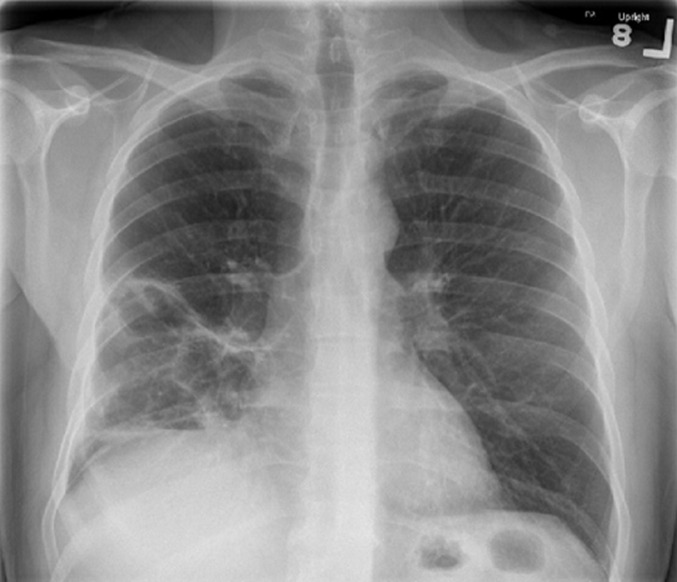
chest X-ray after drainage with a chest tube demonstrated significant improvement in lung aeration with residual pleural and basilar lung changes

Cavitary pneumonia with a fungal pathogen and lung malignancy were other possibilities, but no significant risk factors, such as prolonged neutropenia, chronic steroid use or prior structural lung disease were present. The patient was started on linezolid and meropenem. High-flow oxygen was used to achieve acceptable oxygen saturation level. Repeat chest X-rays on day one, two and three are shown in Figure 2. A repeat CT scan was obtained on day three to identify the structural lung damage better (Figure 3). He developed progressive respiratory failure requiring intubation and mechanical ventilation. An ultrasound revealed complex pleural effusion (Figure 4). A chest tube was inserted and 1.4L of frank pus was drained. A large air leak was noted from bronchopleural fistula (BPF) due to rupture of the abscess cavity in the pleural space. Pleural fluid analysis showed 457,000 leukocyte per cubic milliliter with 99% neutrophil, pH 6.9, glucose < 10mg/dL, protein 4.3gm/dL and lactate dehydrogenase > 10,000 IU/L. The culture was positive for SAG, species *S constellatus*. The patient was treated with ceftriaxone with subsequent improvement. He got liberated from mechanical ventilation. The BPF resolved, and the chest tube was successfully removed (Figure 5). He was completely functional during his outpatient visit a month later.

## Discussion

Rapidly evolving necrotizing pneumonia (NP) complicated by lung abscess and empyema formation is a rare complication of community-acquired bacterial pneumonia. Even though it is rare, the disease progression can be extremely rapid with the virtual destruction of the lung parenchyma in a matter of days. In most reported cases, Panton-Valentine leukocidin (PVL) toxin-producing *S. aureus* (both methicillin sensitive and resistant SA) was found to be the culprit organism [1-3]. The incidence of community-acquired pneumonia (CAP) due to SA is only about 1.6% among all cases of CAP [4]. However, necrotizing pneumonia, especially in the setting of co or prior influenza infection is predominantly due to superimposed SA infection. Viruses other than influenza, such as rhinovirus and respiratory syncytial virus have been shown to increase upper airway colonization by SA transiently, but whether these viruses make the patient more susceptible to SA pneumonia, is unclear [5]. The other reported bacteria include *Streptococcus pneumoniae, Klebsiella pneumoniae*, group A *Streptococcus* and *Pseudomonas aeruginosa* [6-9]. In clinical practice, patients from the community who present with NP, SA infection is almost universally considered to be the likely etiology.

SAG is part of the human oral and gastrointestinal tract microflora and an unusual pathogen for NP. Identification of members of SAG as the causative organism carries important clinical implications. SAG has been implicated in the causation of a broad spectrum of infections. Members of SAG are often associated with pharyngitis and endodontic infections [10]. Suppurative infection, either local or distant, is a common complication. Local suppurative infection in the form of head and neck abscess, jugular vein thrombophlebitis [11], peritonsillar abscess [12] and cervical necrotizing fasciitis [13], have been described. SAG bacteremia in the absence of any apparent infection has also been reported [14]. Identification of SAG bacteremia should prompt a workup for metastatic abscesses [15]. In contrast to NP, due to SA, patients with SAG pneumonia appear less toxic, at least on presentation. Very high fever is usually uncommon [16]. Risk factors include male sex, alcoholism, cancer and cystic fibrosis. Our patient had a co-existent coronavirus infection. Whether that played a role in the pathogenesis of the disease is uncertain. SAG causes invasive lung infection either alone or as a part of polymicrobial infection, including oral anaerobes. Aspiration of oropharyngeal content is the likely inciting event.

Necrotizing pneumonia, lung abscess, empyema, mediastinitis resulting in septic shock and death have been reported [17,18]. SAG has the unique ability to extend beyond fascial planes and has been reported to cross interlobar fissures, diaphragm or even erode through the chest wall [19] and should be considered as a potential etiology in such situations. Management of thoracic infection due to SAG is quite different from the norm. In general, there is no established surgical guideline regarding the management of acute bacterial necrotizing pneumonia. Surgical intervention is usually considered in the setting of septic shock as a source control measure [20]. Lung abscesses that develop from necrotizing pneumonia, communicate with an airway and undergo auto-drainage and usually only require long term antibiotic therapy. But due to the SAG´s ability to cross fascial planes, the lung abscess is often complicated by empyema due to rupture of the abscess in the pleural cavity. In one study, 67% of patients failed antibiotic therapy and required surgical intervention for definitive management [21]. The progression of the disease could be explosive, as was in this case. Four weeks of an intravenous antibiotic such as ceftriaxone with anaerobic coverage or a combination of beta-lactam and a beta-lactamase inhibitor, in addition to surgical intervention is considered optimal therapy.

## Conclusion

Necrotizing pneumonia in the modern era of antibiotic is a rare event. Most common causes include SA or a pneumococcal lung infection. Invasive lung infection due to SAG is a known but underappreciated etiology. SAG should be considered in patients with a fulminant necrotizing lung infection in the presence of risk factors as well as evidence of extension beyond fascial planes. Treatment with antibiotic therapy often fails and surgical intervention should be considered early in the disease process as a delay in therapy could result in significant morbidity and mortality.
